# Professor Barry V.L. Potter: Winner of the 2018 Tu Youyou Award in Honor of the Co-Recipient of the 2015 Nobel Prize in Physiology or Medicine for Her Discoveries Concerning a Novel Therapy against Malaria

**DOI:** 10.3390/molecules23071651

**Published:** 2018-07-06

**Authors:** Derek J. McPhee

**Affiliations:** Editor-in-Chief of *Molecules*; Senior Director, Technology Strategy, Amyris, Inc., 5885 Hollis St, Suite 100, Emeryville, CA 94608, USA; mcphee@mdpi.com

On behalf of MDPI, its flagship journal *Molecules* and its Editorial Board and the Award Selection Committee, whose diligent work evaluating the almost 80 outstanding quality nominations received this year for the award is gratefully acknowledged, it is with great pleasure that I announce that the winner of the 2018 TU YOUYOU AWARD, sponsored by MDPI, is PROFESSOR BARRY V L POTTER, currently Professor of Medicinal and Biological Chemistry at the University of Oxford and Fellow of University College, Oxford, in the United Kingdom. As the awardee, Professor Potter will receive an honorarium of 3000 CHF and an engraved plaque, to be presented by a representative of MDPI and *Molecule*s at a future conference. 

Professor Potter is being recognized not only for his outstanding contributions to Medicinal Chemistry and Chemical Biology through research with a strong relationship to Natural Products Chemistry, but also for his academic excellence, leadership and entrepreneurship across a wide spectrum of activities related to Chemistry, Biology and Medicine. His research interests cover a broad range, from enzyme mechanisms, cellular signaling molecules, the chemistry of nucleic acids, nucleotides and biophosphates to drug design and discovery, where several of his drugs have gone on to “first in class” Phase I and II clinical trials in the areas of breast, prostate and endometrial cancer, endometriosis and hormone replacement therapy. His excellence is exemplified by the many “firsts” in his professional career. Early in his academic career, as a Lecturer in Biological Chemistry at the University of Leicester (equivalent to an Assistant Professor in the U.S. academic system), he was the only chemist to receive a Lister Institute for Preventive Medicine Fellowship for his work on cellular signaling and the mechanisms of RNA splicing. In 1990, he moved to the University of Bath, being unusually directly promoted from Lecturer to Lister Institute Research Professor (equivalent to U.S. Full Professor). At Bath, he would build and lead for over 25 years a globally recognized center of excellence in Medicinal Chemistry. In 2015, he moved to his current position at the University of Oxford where he founded a new Medicinal Chemistry and Drug Discovery Section. He has published over 500 papers and is listed as co-inventor on more than 700 patent applications, over 400 of which have been granted to date. Several of his discoveries form the basis of tools (some of them now part of commercial kits) widely used in pharmacology and chemical biology. As an entrepreneur, he co-founded the University of Bath—Imperial College London spin-off company Sterix Ltd. (subsequently acquired by the French pharma company Ipsen Group), built around his seminal work in the field of steroid sulfatase inhibitors, which includes the design of the anti-breast cancer drug Irosustat.

With these and his many other accomplishments, including election to the UK Academy of Medical Sciences and the pan-European Academia Europaea, there can be no doubt that Professor Potter more than meets the MDPI Tu Youyou Award criteria of authorship of groundbreaking research and significant contributions to the advancement of the natural product and medicinal chemistry fields, and we are greatly honored to add this modest award to his many other recognitions in the fields of medicinal and biological chemistry and pharmacology. 

